# A SNP of *betaine aldehyde dehydrogenase* (*BADH*) enhances an aroma (2-acetyl-1-pyrroline) in sponge gourd (*Luffa cylindrica*) and ridge gourd (*Luffa acutangula)*

**DOI:** 10.1038/s41598-022-07478-9

**Published:** 2022-03-08

**Authors:** Chatree Saensuk, Saowalak Ruangnam, Mutiara K. Pitaloka, Reajina Dumhai, Sugunya Mahatheeranont, Simon Jan de Hoop, Conrado Balatero, Kanamon Riangwong, Vinitchan Ruanjaichon, Theerayut Toojinda, Apichart Vanavichit, Samart Wanchana, Siwaret Arikit

**Affiliations:** 1 Interdisciplinary Graduate Program in Genetic Engineering, Kasetsart University, Bangkok, 10900 Thailand; 2 Hortigenetics Research (S.E. Asia) Limited, Suphan Buri, 72190 Thailand; 3 Rice Science Center, Kasetsart University Kamphaeng Saen Campus, Nakhon Pathom, 73140 Thailand; 4 Department of Chemistry, Faculty of Science, Chiang Mai University, Chiang Mai, 50200 Thailand; 5 Department of Biotechnology, Faculty of Engineering and Industrial Technology, Silpakorn University, Sanamchandra Palace Campus, Nakhon Pathom, 73000 Thailand; 6 National Center for Genetic Engineering and Biotechnology (BIOTEC), National Science and Technology Development Agency (NSTDA), Khlong Luang, 12120 Pathum Thani Thailand; 7 Department of Agronomy, Faculty of Agriculture at Kamphaeng Saen, Kasetsart University Kamphaeng Saen Campus, Nakhon Pathom, 73140 Thailand

**Keywords:** Plant breeding, Plant sciences, Plant biotechnology, Agricultural genetics

## Abstract

*Luffa* is a genus of tropical and subtropical vines belonging to the Cucurbitaceae family. Sponge gourd (*Luffa cylindrica*) and ridge gourd (*Luffa acutangula*) are two important species of the genus *Luffa* and are good sources of human nutrition and herbal medicines. As a vegetable, aromatic luffa is more preferred by consumers than nonaromatic luffa. While the aroma trait is present in the sponge gourd, the trait is not present in the ridge gourd. In this study, we identified *Luffa cylindrica*’s *betaine aldehyde dehydrogenase* (*LcBADH*) as a gene associated with aroma in the sponge gourd based on a de novo assembly of public transcriptome data. A single nucleotide polymorphism (SNP: A > G) was identified in exon 5 of *LcBADH*, causing an amino acid change from tyrosine to cysteine at position 163, which is important for the formation of the substrate binding pocket of the BADH enzyme. Based on the identified SNP, a TaqMan marker, named *AroLuff*, was developed and validated in 370 F_2_ progenies of the sponge gourd. The marker genotypes were perfectly associated with the aroma phenotypes, and the segregation ratios supported Mendelian’s simple recessive inheritance. In addition, we demonstrated the use of the *AroLuff* marker in the introgression of *LcBADH* from the aromatic sponge gourd to the ridge gourd to improve aroma through interspecific hybridization. The marker proved to be useful in improving the aroma characteristics of both *Luffa* species.

## Introduction

*Luffa* is a genus of tropical and subtropical vines classified as Cucurbitaceae. Sponge gourd [*Luffa cylindrica* (L.) Roem, syn. *L. aegyptica* Mill] and ridge gourd [*Luffa acutangula* (L.) Roxb.] are two important species under the genus *Luffa* commonly cultivated for their fruits, which are edible when young and have a fibrous sponge-like texture when mature. Both the sponge gourd and the ridge gourd are good sources of various minerals, carbohydrates, phosphorus, and vitamin C, which makes them important vegetables and good for human nutrition^1,2^. These gourds are also known for their medicinal function. Some tissues, such as leaves, seeds and fruits, are used in the treatment of various diseases, especially diabetes, inflammatory diseases, diarrhea, and viral infections^3,4^.

The sponge gourd and the ridge gourd are important vegetables widely grown in tropical and subtropical countries. A special type of sponge gourd with a pleasant “pandan-like” aroma is also available in some countries, such as Thailand and Vietnam. Aromatic sponge gourd is more preferred by consumers than nonaromatic sponge gourd. Unlike the sponge gourd, there is no report of the existence of an aromatic type of ridge gourd. Aroma is a value-added trait in several food crops, such as rice and vegetable soybean^5,6^. Products with aroma have a higher demand and can achieve a higher price than products without aroma^6^. The 2-acetyl-1-pyrroline (2AP) is generally believed to be the main component responsible for the “popcorn-like” or “pandan-like” aroma of plants^7^. A variety of plants are known to synthesize 2AP, including pandan (*Pandanus amaryllifolius* Roxb.)^8^, rice (*Oryza sativa* L.)^5,8–10^, bread flowers (*Vallaris glabra* Ktze)^11^, soybean (*Glycine max* L.)^12,13^, sorghum (*Sorghum bicolor* L.)^14^, cucumber (*Cucumis sativus* L.)^15^, *Bassia latifolia* Roxb^16^, winter melon (*Benincasa hispida*)^17^ and coconut (*Cocos nucifera*)^18,19^.

The gene that plays an important role in the 2AP biosynthetic pathway of crops was first identified in rice^20^ and named *BAD2* or *BADH2* based on sequence similarity to the previously identified *betaine aldehyde dehydrogenase* (*BADH*). Later, the gene was synonymized to *aminoaldehyde dehydrogenase* (*AMADH*), a member of plant ALDH10 that catalyzes a whole range of aminoaldehydes^12,21^. The functional BADH/AMADH catalyzes the oxidation of gamma-aminobutyraldehyde, which is the substrate of 2AP, leading to the synthesis of gamma-aminobutyric acid (GABA). The loss of *BADH2/AMADH2* function leads to the accumulation of gamma-aminobutyraldehyde, which is subsequently converted to 2AP^20^. The study of aroma genes in crop plants is then gradually expanding, as orthologs of this gene have been characterized as being responsible for aroma phenotypes in several crops, i.e., soybean^12,22^, sorghum^14^, and crops in the Cucurbitaceae family, such as cucumber^15^ and winter melon^17^. The study of the gene has also extended to tree plants such as coconut^18,19^. Several allelic variations have also been reported for this gene in several plants, such as in rice^23,24^, soybean^12,22^ and coconut^18,19^.

Although the gene and molecular mechanism associated with 2AP biosynthesis have been well elucidated in various crops, the detection of the gene and molecular mechanism has not been reported in *Luffa*, making it difficult to improve an elite variety with the aroma trait. In this study, we identified the gene associated with aroma in the sponge gourd (*Luffa cylindrica*) based on public transcriptome analysis. In addition, we developed a functional marker based on the SNP identified in the gene, validated it in an F_2_ population of sponge gourd, and used it to select the desired plants in an interspecific population (sponge gourd × ridge gourd). The marker proved useful in improving the aroma trait of *Luffa*.

## Results

### Confirmation of 2AP as a potent volatile compound contributing to the aroma of aromatic sponge gourd

The aromatic sponge gourd (*Luffa cylindrica*) has a “popcorn-like” or “pandan-like” aroma that is absent in its nonaromatic counterpart. As reported in other plants, this aroma is due to the potent volatile compound 2-acetyl-1-pyrroline (2AP). To confirm that the aroma in sponge gourd is also due to 2AP, we analyzed the 2AP content in the fruit from an aromatic inbred line, PB-00493, and a nonaromatic inbred line, PB-00492, using gas chromatography/mass spectrometry (GC/MS). As expected, 2AP was detected at a high concentration (7.33 ppm) in PB-00493, while it was found at a negligible level (0.39 ppm) in PB-00492 (Supplementary Fig. S1). This result indicates that the aroma of the aromatic sponge gourd is due to the presence of 2AP.

### Identification of the *BADH2* ortholog in sponge gourd based on a de novo transcriptome assembly

The key gene responsible for 2AP in plants is described as a nonfunctional version of *betaine aldehyde dehydrogenase 2 (BADH2),* also called *aminoaldehyde dehydrogenase* (*AMADH)*^12,15,17–19^. We hypothesized that the production of 2AP in the sponge gourd is also linked to *BADH2*. We attempted to identify the gene using public transcriptome data, which we assembled into a de novo transcript assembly using Trinity. As a result, 63,392 transcript contigs were assembled, of which 50,146 contained coding sequences (CDSs) and 21,977 had a BLAST hit (Supplementary Table S1). We then used the winter melon *BhAMADH*^17^ as a search query (tblastn similarity search) against these assembled contigs to identify the *BADH2* ortholog in sponge gourd. As a result, a 2235-bp-long transcript contig (c16191_g1_i1) was identified as the best match, with 94.43% identity to the winter melon query gene at the amino acid level (Supplementary Fig. S2). This candidate has a 1512 bp long coding sequence (CDS) that can be translated into 503 amino acids (Supplementary Fig. S3). The gene has been named and will be referred to as *LcBADH* hereafter.

### *LcBADH* gene structure and sequence variation

**Figure 1:**
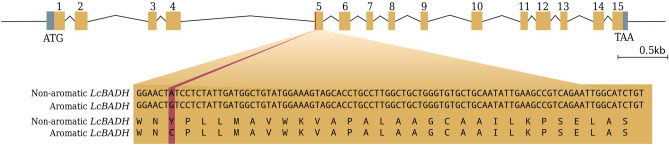
Structure of *LcBADH* and sequence variation of the gene showing the single nucleotide change on exon 5 associated with the aromatic phenotype on sponge gourd. The exons of the gene model are shown in a brown highlighted rectangle and the introns are represented by thin lines. The gray highlighted rectangles at the beginning and end of the gene model indicate the 5′ and 3′ UTR regions. The base substitution and amino acid change in exon 5 are shown in red.

**Figure 2:**
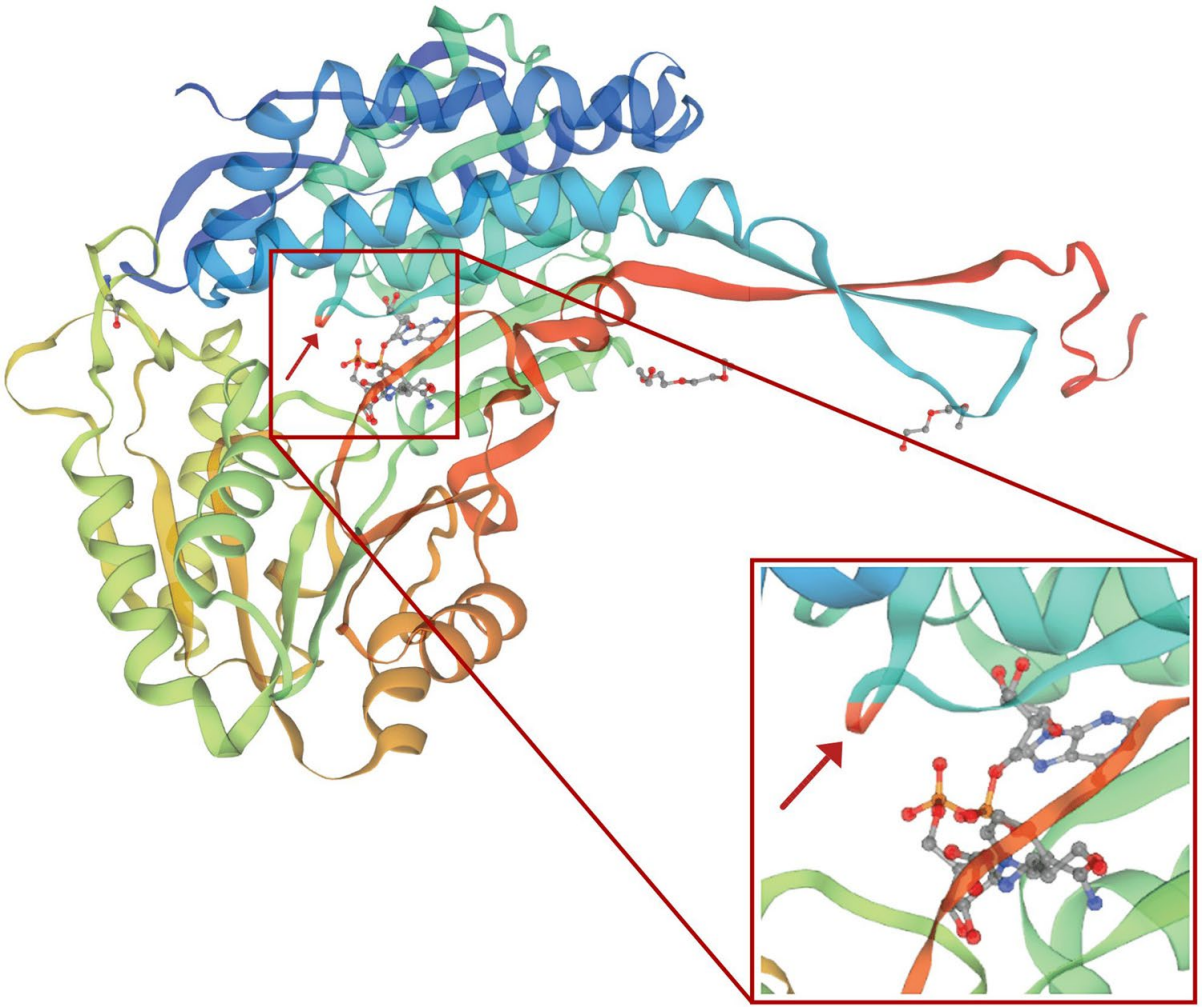
Three-dimensional protein structure homology models of LcBADH visualized using PyMOL (The PyMOL Molecular Graphics System, Version 2.5 Schrödinger, LLC). The expanded box shows the cysteine mutant at position 163 as indicated by a red arrow.

The structure of *LcBADH* was characterized by alignment of transcript contig c16191_g1_i1 with the cDNA sequence and genomic DNA sequence of *BhAMADH* from winter melon (*Benincasa hispida*). As a result*, LcBADH* was annotated with 15 exons separated by 14 introns (Supplementary Fig. S4). To determine the sequence variation in the *LcBADH* gene, we first sequenced the entire gene and then aligned the sequences comparing an aromatic (PB-00493) and a nonaromatic (PB-00492) sponge gourd inbred line. To sequence the entire *LcBADH*, we used a set of primers to amplify the genomic DNA of the sponge gourd inbred lines. The primers were designed to generate DNA fragments covering the entire *LcBADH* gene (Supplementary Fig. S5). The amplified fragments were sequenced using Sanger sequencing, and then the sequences were assembled into a full-length gene and aligned between aromatic and nonaromatic sponge gourd. Sequence alignment revealed a base substitution (A > G) in exon 5 causing a change in the amino acid tyrosine to cysteine (Y > C) at position 163 of the protein sequence in aromatic sponge gourd compared to nonaromatic sponge gourd (Fig. 1). Amino acid Y-163 is one of the six residues predicted to form the substrate binding pocket of the BADH/AMADH enzyme (Fig. 2). In addition, this position is near N-162, one of the two catalytic residues predicted to interact with substrate oxygen^25^.

### *LcBADH* gene expression in various tissues of aromatic and nonaromatic sponge gourd

**Figure 3:**
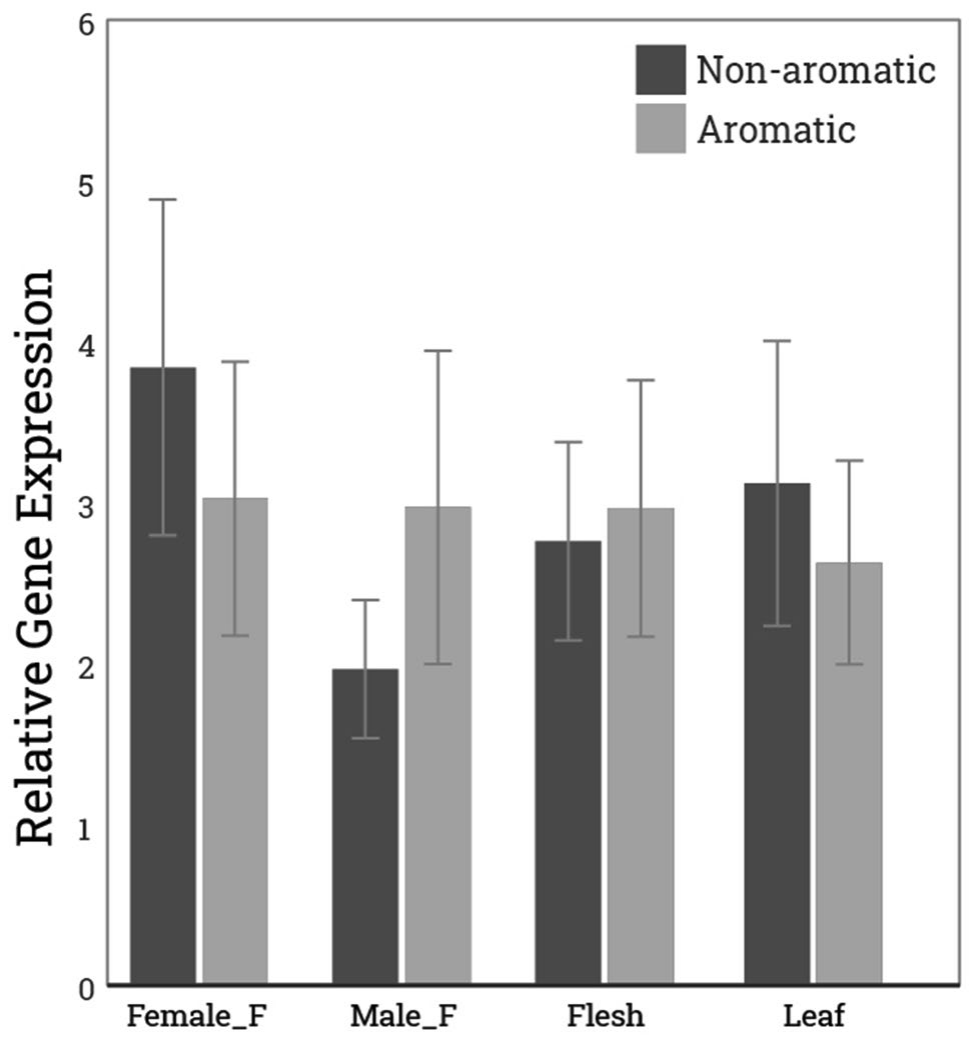
Relative normalized expression of *LcBADH* based on qRT-PCR in female flower, male flower, fruit, and leaf tissues compared between nonaromatic sponge gourd (PB-00492) and aromatic sponge gourd (PB-00493). Dark gray represents nonaromatic sponge gourd and pale gray represents aromatic sponge gourd. The I bars represent the standard deviation.

To verify whether the SNP found in *LcBADH* affects gene expression, we performed quantitative RT-PCR (qRT-PCR) to analyze gene expression in different tissues (male and female flowers, leaves and fruits) and compare aromatic and nonaromatic sponge gourds. The results of qRT-PCR performed on three biological replicates showed that there was no significant difference in *LcBADH* gene expression when comparing aromatic and nonaromatic sponge gourd (Fig. 3), suggesting that the SNP does not affect gene expression. However, since the sequence variation in exon 5 in aromatic sponge gourd resulted in a change in the amino acid C163Y, which is important for enzyme activity, this mutation may cause a change in enzyme activity. Consequently, 2AP is enhanced.

### A functional marker of the aroma of sponge gourd

Based on the single nucleotide polymorphism (A/G) in exon 5 of *LcBADH*, we developed a TaqMan probe that can detect three genotypes: homozygous G/G, heterozygous A/G, and homozygous A/A (Table 1). The marker is hereafter referred to as *AroLuff*. To evaluate the efficacy of the *AroLuff* marker for the aroma trait in sponge gourd, we genotyped 370 F_2_ individuals derived from a cross between aromatic sponge gourd (PB-00493) and nonaromatic sponge gourd (PB-00492) and evaluated the aroma phenotype in this population using a sensory test. The genotyping results showed that 87 plants had the homozygous (G/G) genotype, 189 plants had the heterozygous (A/G) genotype, and 94 plants had the homozygous (A/A) genotype (Supplementary Fig. S6). Based on the sensory test results, all homozygous (G/G) plants were classified as aromatic, while those homozygous (A/A) and heterozygous (A/G) plants were classified as nonaromatic (Supplementary Table S2). We also performed a chi-square test for the fit of the segregating phenotypes and segregating genotypes of 370 F_2_ individuals. The results showed that both ratios perfectly matched the Mendelian ratio for a single recessive gene controlling the trait (Table 2). Based on these results, we confirmed with certainty that *LcBADH* is the key gene responsible for the aroma trait (2AP) in sponge gourd.

**Table 1:** Primer and probe sequences for AroLuff TaqMan marker.

Primer/probe name	Sequence (5′–3′)
Forward primer	CAAACGTTGCTGATGTCTGTCTTTT
Reverse primer	CAAGGCAGGTGCTACTTTCCA
Reporter 1 (VIC)	ATCAATAGAGGA**T**AGTTCC
Reporter 2 (FAM)	CAATAGAGGA**C**AGTTCC

**Table 2:** Chi-square test of aroma phenotype and genotypes.

	Number		*χ*^2^	*P* value
	Observed	Expected		
**Phenotype (expected ratio is 1:3)**				
Aroma	87	92.5		
Non aroma	283	277.5		
Total	370		0.4360	0.5090
**Genotype (expected ratio 1:2:1)**				
A/A	94	92.5		
A/G	189	185		
G/G	87	92.5		
Total	370		0.4378	0.8034

### Transfer of *LcBADH* from aromatic sponge gourd to ridge gourd through an interspecific hybridization and selection of the aroma using the *AroLuff* marker

**Figure 4:**
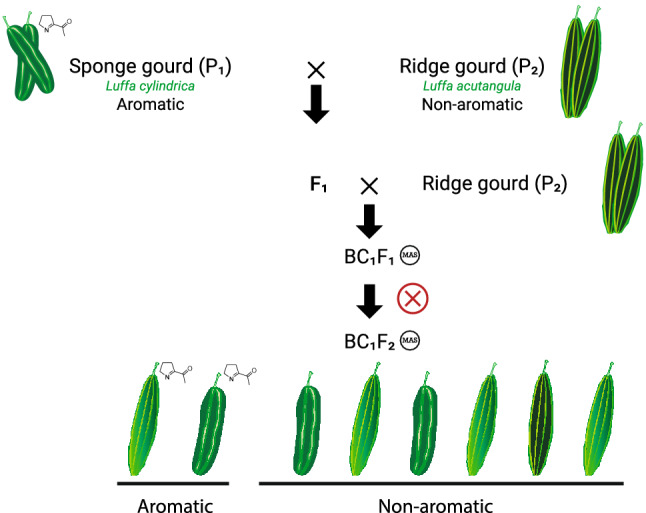
Scheme of interspecific breeding of aromatic ridge gourd.

Ridge gourd [*Luffa acutangula* (L.) Roxb.] is another luffa species that is also an important vegetable grown in tropical countries. To date, no aromatic ridge gourd variety has been described. Having successfully demonstrated that *LcBADH* is a key gene controlling the aroma trait in sponge gourd (*L. cylindrica*), we sought to introduce the gene from aromatic sponge gourd into ridge gourd (*L. acutangula*) to improve the aroma in the latter species. First, we crossed the aromatic sponge gourd (PB-00493) with ridge gourd (PB-00491) to produce F_1_ plants (PB-00493 × PB-00491). Then, F_1_ plants (*L. cylindrica* × *L. acutangula*) were used as the male parent for backcrossing with ridge gourd (PB-00491) to produce BC_1_F_1_ plants [PB-00491 × (PB-00493 × PB-00491)] (Fig. 4). We used the *AroLuff* marker to genotype BC_1_F_1_ plants to select plants with the heterozygous A/G genotype. Of the 66 BC_1_F_1_ plants, 16 were selected and self-pollinated to produce the BC_1_F_2_ generation. A total of 93 BC_1_F_2_ plants were generated. These plants were genotyped with the *AroLuff* marker, and the aroma of their fruits was evaluated with a sensory test (Supplementary Table S3). The results showed that all plants with genotype G/G were aromatic, while those with heterozygous A/G and homozygous A/A were not.

**Figure 5:**
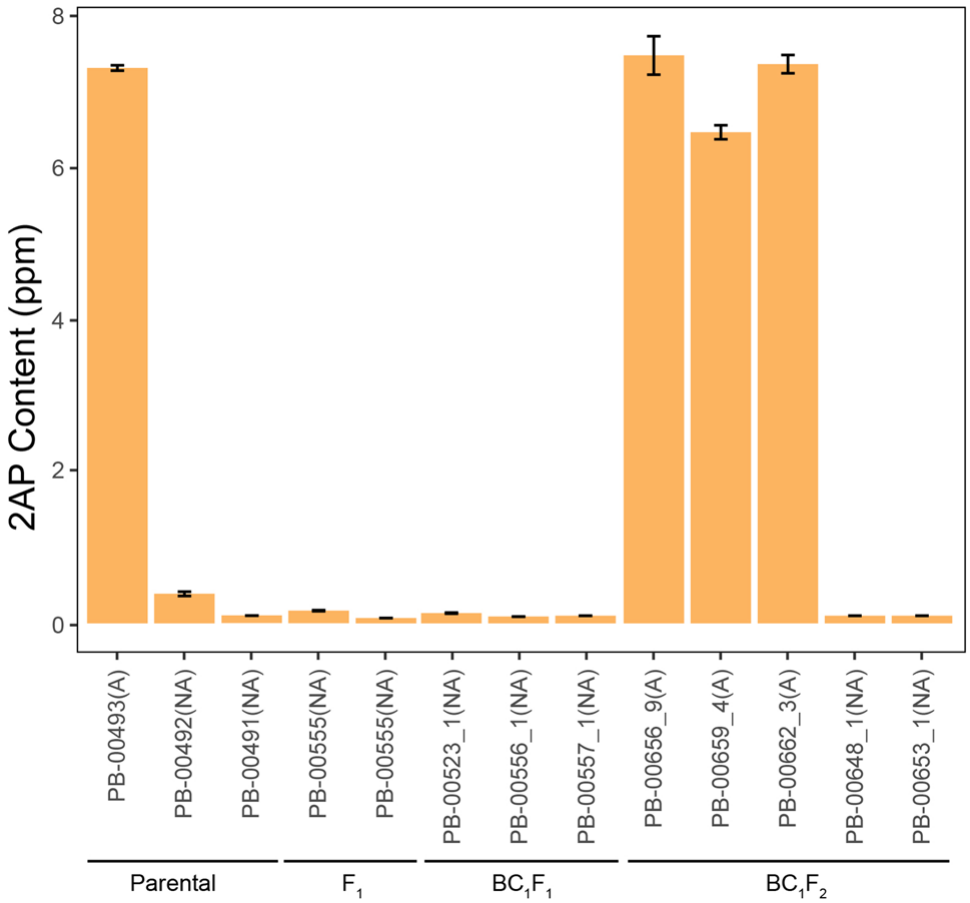
2AP analysis in parental lines and different generations of interspecific crosses. The letters A and NA after the plant names indicate aromatic and nonaromatic phenotypes, respectively.

To confirm that 2AP was produced and accumulated in the plants with genotype G/G, we analyzed the 2AP content in the fruits of some representatives of the interspecific cross in all generations, i.e., F_1_, BC_1_F_1_, and BC_2_F_2_, compared with the aromatic sponge gourd parent (PB-00493). As a result, a high 2AP content was observed in the aromatic plants with genotype G/G in all generations (Fig. 5).

### Synteny analysis of *BADH* orthologous genes in some Cucurbitaceae crops

**Figure 6:**
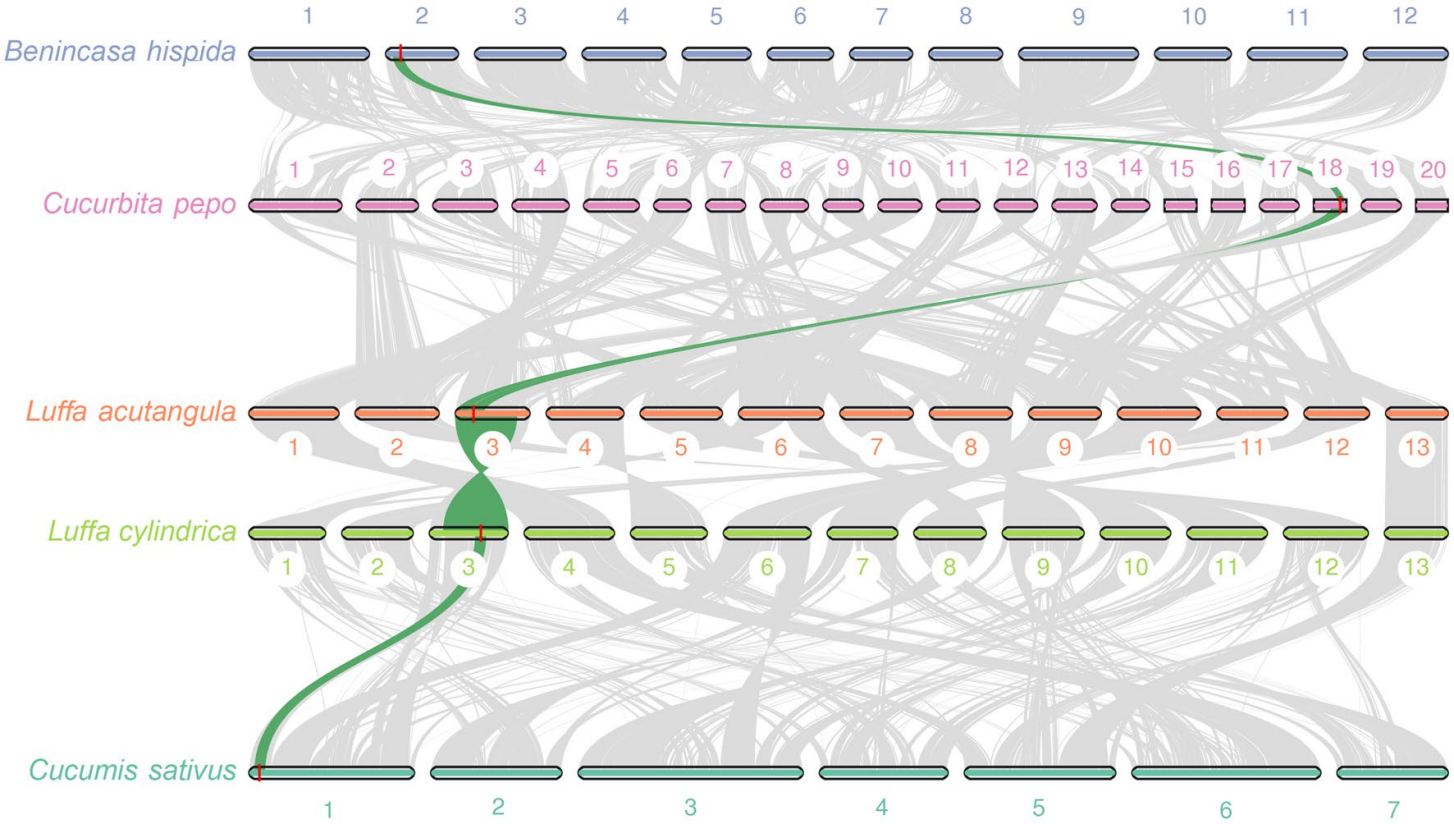
Syntenic relationship of *BADH* genes among *Benincasa hispida, Cucurbita pepo, Luffa acutangula, Luffa cylindrica* and *Cucumis sativus* visualized using MCscan (Python version (https://github.com/tanghaibao/jcvi/wiki/MCscan-(Python-version))*.* The green line indicates the synteny block and the red mark indicates the gene position.

Interspecies synteny block identification was calculated using the pairwise MCScanX approach to identify *BADH* synteny blocks among species of Cucurbitaceae, including *Benincasa hispida* (2n = 24), *Cucurbita pepo* (2n = 40), *Luffa acutangula* (2n = 26), *Luffa cylindrica* (2n = 26) and *Cucumis sativus* (2n = 14). In these species, only one copy of *BADH* was found in their genome. The *BADH* gene was located on chromosome 3 in both Luffa species, whereas it was found on chromosome 2 in *B. hispida*, chromosome 18 in *C. pepo,* and chromosome 1 in *C. sativus* (Fig. 6). Based on the protein sequence alignment of the BADH orthologs of these species, we found high protein sequence similarity (94.83–100% similarity: Supplementary Table S6). The BADH protein sequences of both *Luffa* species are identical. Moreover, all major conserved residues for the catalytic sites of ALDH10 family enzymes were found to be identical in these species (Supplementary Fig. S7). The amino acid change C163Y, found exclusively in the aromatic sponge gourd, is located adjacent to the conserved N-162 residue.

## Discussion

Luffa crops, sponge gourd (*Luffa cylindrica*) and ridge gourd (*Luffa acutangula*), are important vegetables in the Cucurbitaceae family. Like other crops, aromatic luffa with a pleasant aroma is preferred on the market because it has higher quality and acceptability and is preferred by consumers. Therefore, the aroma trait is attractive for breeders to include in the pipeline of the improvement of elite varieties. The volatile compound 2-acetyl-1-pyrroline (2AP) is known as the main component of the “popcorn-like” or “pandan-like” aroma in plants^5,6,8,10,11,14,15,17–19^. In this study, we confirmed that this compound is also a major component of aroma in aromatic sponge gourd. To date, *BADH2* is the only major gene associated with 2AP biosynthesis in plants^7^. In this study, we identified and confirmed that *LcBADH* is also the key gene for the aroma trait in sponge gourd. Comparing aromatic and nonaromatic sponge gourd, the gene differed by a single nucleotide change in exon 5. Sequence variations, both SNPs and indels, identified in the coding sequence of *BADH2* resulted in inactivation of BADH/AMADH enzyme activity and promoted 2AP biosynthesis^5,12,25^. Sequence mutations may or may not affect gene expression. For example, the 8-bp deletion in exon 7 of *OsBADH2* in rice, the 2-bp deletion in exon 10 of *GmAMADH2*, and the 804-bp deletion in *BhAMADH* of winter melon resulted in a decrease in gene expression^5,12,17^. In contrast, the SNPs in exons 9 and 14 of *CnAMADH2* in coconut had no effect on gene expression^18,19^. In this study, the expression of *LcBADH* did not differ between aromatic and nonaromatic sponge gourd according to qRT-PCR results. Thus, the mutation in exon 5 *LcBADH* had no effect on gene expression. However, because this mutation occurs at a position necessary for the BADH enzyme activity, we assumed that the mutation would affect protein function. Nevertheless, further study on an enzymatic assay is needed to confirm this.

Functional markers for the aroma gene have been developed for use in breeding programs in several plants ^6,14,15,17,19,22^. In this study, we developed the *AroLuff* marker based on the SNP in exon 5 of *LcBADH* and validated this marker in an F_2_ population segregating for aroma. The genotypes of the marker were perfectly associated with the aroma phenotypes. Segregation of phenotypes and genotypes in this population perfectly supported Mendelian inheritance of a single recessive gene for the aroma trait in sponge gourd. The *AroLuff* marker is not only useful for improving the aroma trait in sponge gourd but can also be used to improve the trait in ridge gourd. Ridge gourd and sponge gourd are two related species that have similar genome sizes (~ 760–790 Mb) and the same number of chromosomes (2n = 2x = 26). Since the aroma trait is not available in ridge gourd, we tried to transfer the trait from sponge gourd to ridge gourd by interspecific hybridization. We successfully used the *AroLuff* marker to select plants containing the aromatic allele of *LcBADH* in interspecific backcross populations of the BC_1_F_1_ and BC_1_F_2_ generations. We also confirmed that BC_1_F_2_ plants containing the homozygous recessive genotype of *LcBADH* are aromatic and produce 2AP at high levels. This is the first report in which the aroma trait is transferred from one species to a closely related species by interspecific hybridization combined with marker-assisted selection.

In many plant lineages, two copies of the *BADH/AMADH* genes, *BADH1/AMADH1* and *BADH2/AMADH2*, are present in the genome^12,18,19^. In rice and soybean, the two copies of *BADH/AMADH* were thought to be involved in different metabolic pathways^5,12^. Functional *BADH1/AMADH1* is involved in the glycine-betaine biosynthetic pathway, whereas functional *BADH2/AMADH2* is involved in the gamma aminobutyric acid (GABA) synthetic pathway. Most studies have reported that nonfunctional *BADH2/AMADH2* is the cause of 2AP biosynthesis^5,12,18,19,22,25^. However, there have also been a few reports on the relationship between *BADH1* and 2AP^26,27^. Comparing the genome synteny of five species of Cucurbitaceae, we found that all these species contain only one copy of *BADH/AMADH*. Mutations in *BADH/AMADH* were reported in *Benincasa hispida* and *Cucumis sativus* and were associated with 2AP biosynthesis^15,17^. In the present study, we reported a mutation in *LcBADH* and its association with 2AP biosynthesis in *Luffa cylindrica*. Therefore, it is possible that a mutation in the *BADH* gene on chromosome 18 of *Cucubita pepo*, if present, could also be associated with 2AP.

Interspecific hybridization, which involves crossing two species from the same genus, could be used to improve traits in one species by using useful genes from a closely related species or from wild species, which has helped in the introgression of important traits in many vegetable crops^28,29^. The nearly perfect genome synteny between *L. cylindrica* and *L. acutangula* supported our success in interspecific hybridization between the two species. Our study clearly demonstrated that the aroma trait can be transferred between the two *Luffa* species, and the *AroLuff* marker will be useful in breeding programs to improve aroma in both luffa crops.

## Conclusion

The gene conferring the “pandan-like” aroma trait in sponge gourd has been characterized. A SNP (A > G) found in the aromatic allele of the *LcBADH* gene has been shown to be associated with the aroma of sponge gourd. The functional marker “*AroLuff*” developed based on this SNP has proven useful in marker-assisted (MAS) breeding for the aroma trait in ridge gourd through interspecific hybridization. This is the first time that the aroma trait has been transferred between the two closely related species.

## Materials and methods

### Plant materials

Three hundred and seventy F_2_ individuals derived from the seeds of 15 F_1_ plants of a cross between an aromatic (PB-00493) and a nonaromatic (PB-00492) inbred line of sponge gourd (*Luffa cylindrica*) were used to evaluate aroma and validate the marker. An inbred line of ridge gourd (*Luffa acutangula*: PB-00491) was used to produce interspecific F_1_ progeny by crossing with aromatic sponge gourd (PB-00493) and used as a recurrent parent to produce BC_1_F_1_ progeny. Sixty-six BC_1_F_1_ plants were genotyped with the marker to select the plants with a heterozygous genotype in the gene *LcBADH*, and the selected plants were then self-pollinated to produce BC_1_F_2_ progeny. The sponge gourd and ridge gourd cultivars used as parents for the above populations were used together with representatives of F_1_ (PB-00555), F_2_ (PB-00522), BC_1_F_1_ (PB-00557), and BC_1_F_2_ plants to determine the content of 2-acetyl-1-pyrroline (2AP). Aromatic (PB-00493) and nonaromatic (PB-00492) sponge gourds were also used for sequencing the full-length *LcBADH* gene and analysis of gene expression. Field experiments were conducted at the research station of Hortigenetics Research (S.E. Asia) Limited, Suphanburi, Thailand, between July 2017 and November 2018. All plant materials used in the study were provided by Hortigenetics Research (S.E. Asia). All experiments complied with the current Biosafety guidelines of the country in which the experiments were performed.

### Identification of 2AP using gas chromatography/mass spectrometry (GC/MS)

The 2AP analysis was performed on fruit from three F_1_ (PB-00493 × PB-00492), five aromatic, and six nonaromatic representative F_2_ plants, two interspecific F_1_ (PB-00493 × PB-00491) plants, three interspecific BC_1_F_1_, five interspecific BC_1_F_2_ plants, and the parental lines PB-00491 (ridge gourd), PB-00492 (nonaromatic sponge gourd) and PB-00493 (aromatic sponge gourd). The GC/MS system was an Agilent GC 6890 and 6850-MS 5973 (Agilent Technology, Palo Alto, CA). Extraction, detection, and quantification of 2AP from fruit flesh of sponge gourd and ridge gourd were performed according to a method described previously^17^.

### Sensory test for aroma evaluation

The aroma characteristics of 370 F_2_ progenies of sponge gourd and 93 interspecific BC_1_F_2_ were evaluated using a sensory test method modified from^30^. One gram of fresh flesh of the 20-day-old fruit was cut into small pieces and placed in a Petri dish, which was then soaked with 2 ml of 1.7% KOH. The dish was covered with a lid and set aside at room temperature for 10 min. The aroma was then scored by three trained panels. Aroma characteristics were classified as present (aromatic) or absent (nonaromatic). Three fruits from each line were scored as replicates.

### DNA and RNA extraction

Genomic DNA was isolated from young leaves using a DNeasy Plant Mini Kit (Qiagen Inc., Valencia, CA, USA). DNA quality was determined using a NanoDrop 8000 (Thermo Fisher Scientific Inc., MA, USA). DNA was stored at – 20 °C until use. Total RNA for gene expression analysis was extracted from pistils, stamens, leaves and fruits (20 days after pollination) of aromatic and nonaromatic sponge gourd using the RNeasy Plant Mini Kit (Qiagen, Inc.). The quality of total RNA was determined using a NanoDrop 8000 (Thermo Fisher Scientific Inc.). The RNA was stored at − 80 °C until use.

### De novo transcriptome assembly and annotation

Transcriptome sequencing data of *L. cylindrica* were downloaded from the National Center for Bitechnology Information (NCBI)’s Sequence Read Archive (SRA) database (Accession number SRR1023265) and subjected to de novo transcriptome assembly. The data contained 59,874,228 pairs of 150-bp paired-end Illumina sequences. Raw data (fastq) were assessed with FASTQC (http://www.bioinformatics.babraham.ac.uk/projects/fastqc/) and trimmed adapters and low-quality bases with Trimmomatic^31^. The quality cutoff was a PHRED33 score of > 10. Only sequencing reads ≥ 100 bp were retained. Reads that contained a portion with an average PHRED33 score < 10 that spanned at least 4 bp were removed. The clean paired-end Illumina reads were subjected to the Trinity assembly pipeline (Trinity Release v2.2.2) using the default parameters for de novo transcriptome assembly^32^. Assembly results were assessed using the TrinityStats.pl script that comes with Trinity, and contigs were annotated by similarity search (NCBI blastx) against a nonredundant (nr) database using standalone command line BLAST.

### Sequencing of the *LcBADH* gene in aromatic and nonaromatic varieties of sponge gourd

To obtain the full-length sequence of *LcBADH* in aromatic sponge gourds, a set of primers was designed using Primer3^33^ based on the sequence of the assembled contig (Supplementary Table S4). For each primer pair, polymerase chain reaction (PCR) was performed in a total volume of 10 μl containing 2 μl of genomic DNA (50 ng/μl), 1 μl of 10 × buffer, 1 μl of 25 mM MgCl_2_, 2 μl of 1 mM dNTPs, 0.5 μl of each primer (5 μM), and 0.1 μl of Taq DNA polymerase (Fermentas; Life Science, USA). PCR was initiated by denaturation at 95 °C for 3 min followed by 35 cycles at 95 °C for 15 s, 55 °C for 30 s, and 72 °C for 1 min before a final incubation at 72 °C for 10 min to complete primer extension. The amplicons were purified and sequenced using a Sanger sequencing method at First BASE Laboratories Sdn Bhd, Selangor, Malaysia. The sequences of each variety were assembled and aligned using CLC Genomics Workbench (CLC Bio; Qiagen, USA).

### Three-dimensional protein structure modeling

The coding sequence (CDS) of *LcBADH* was translated into amino acid sequences using the Translate tool on the ExPASy website (http://web.expasy.org/translate/). The Swiss-Model Server^34^ (https://swissmodel.expasy.org) was used to create a 3D homology model of the LcBADH protein based on the BADH protein (PDB ID: 3IWJ) from *Pisum sativum* as a template. The quality of the 3D structural models was assessed using PROCHECK version 3.5.4^35^. The 3D models were visualized in PyMOL (The PyMOL Molecular Graphics System, Version 2.5 Schrödinger, LLC).

### Quantitative reverse transcription PCR (qRT-PCR) analysis

qRT-PCRs were performed using iScript One-Step RT-PCR reagent with SYBR Green (Bio-Rad, USA) to analyze the relative expression of the gene in different tissues (male and female flowers, fruit flesh and leaf) of aromatic and nonaromatic sponge gourd. Three biological replicates for aromatic and nonaromatic phenotypes were used. Total RNA at a level of one ng per sample was used as a template, and the β-actin forward primer was used as an internal reference gene to normalize the variations of the total cDNA template between samples. The gene-specific primers and actin primers are listed in Supplementary Table S5. Relative gene expression was analyzed using Bio-Rad CFX Manager analysis software (Bio-Rad, USA) to compare the change in expression compared with β-actin as a control. The results were then statistically analyzed using Student’s *t*-test. A *P* value < 0.05 was considered significant.

### Development of TaqMan assays for *LcBADH* allelic variation detection

TaqMan allele-specific assays (primers and probes) for *LcBADH* were designed and synthesized by Thermo Fisher Scientific (Thermo Fisher Scientific, USA). PCR for the TaqMan® assay was performed in a total volume of 5 µl containing 1.5 μl of genomic DNA (20 ng/µl), 2.5 μl of 2 × GT express, and 0.125 μl of assay probe primer (40x), adjusted to 0.875 μl with ddH_2_O. The cycling conditions were 95 °C for 5 min, 40x [94 °C for 30 s and 60 °C for 1 min] and 60 °C for 2 min. Genotype calling was performed using a QuanStudio™ (Thermo Fisher Scientific, USA).

### Synteny block analysis

Genome assembly data of *Luffa cylindrica* (GCA_017139565.1), *Luffa acutangula* (GCA_012295215.1), *Benincasa hispida* (GCF_009727055.1), *Cucurbita pepo* (GCF_002806865.1) and *Cucumis sativus* (GCF_000004075.3) were obtained from the NCBI database. Synteny blocks were generated using the MCScanX^36^. Syntenic relationships were visualized using Python version of MCscan^37^ (https://github.com/tanghaibao/jcvi/wiki/MCscan-(Python-version)). The region in the genome matching the *BADH* gene was searched using the MCScanX^36^.

## Supplementary Information

Supplementary Information.

## Data Availability

Transcriptome data can be found in the NCBI-SRA database, with the accession number SRR1023265. The genome assembly data of *Luffa cylindrica* (GCA_017139565.1), *Luffa acutangula* (GCA_012295215.1), *Benincasa hispida* (GCF_009727055.1), *Cucurbita pepo* (GCF_002806865.1) and *Cucumis sativus* (GCF_000004075.3) were obtained from the NCBI database.
