# Supplementary material for: A SNP of betaine aldehyde dehydrogenase (BADH) enhances an aroma (2-acetyl-1-pyrroline) in sponge gourd (Luffa cylindrica) and ridge gourd (Luffa acutangula)
Source: Sci Rep. 2022 Mar 8;12:3718. doi: 10.1038/s41598-022-07478-9 (PMC8904516; doi:10.1038/s41598-022-07478-9)
Supplement: Supplementary file 1 — Supplementary Information. [file 41598_2022_7478_MOESM1_ESM.pdf]

Supplementary Information

A SNP of betaine aldehyde dehydrogenase (BADH) enhances an aroma (2-acetyl-1-pyrroline) in sponge gourd (*Luffa cylindrica*) and ridge gourd (*Luffa acutangula*)

Chatree Saensuk, Saowalak Ruangnam, Mutiara K. Pitaloka, Reajina Dumhai, Sugunya Mahatheeranont, Simon Jan De Hoop, Conrado Balatero, Kanamon Riangwong, Vinitchan Ruanjaichon, Theerayut Toojinda, Apichart Vanavichit, Samart Wanchana & Siwaret Arikit

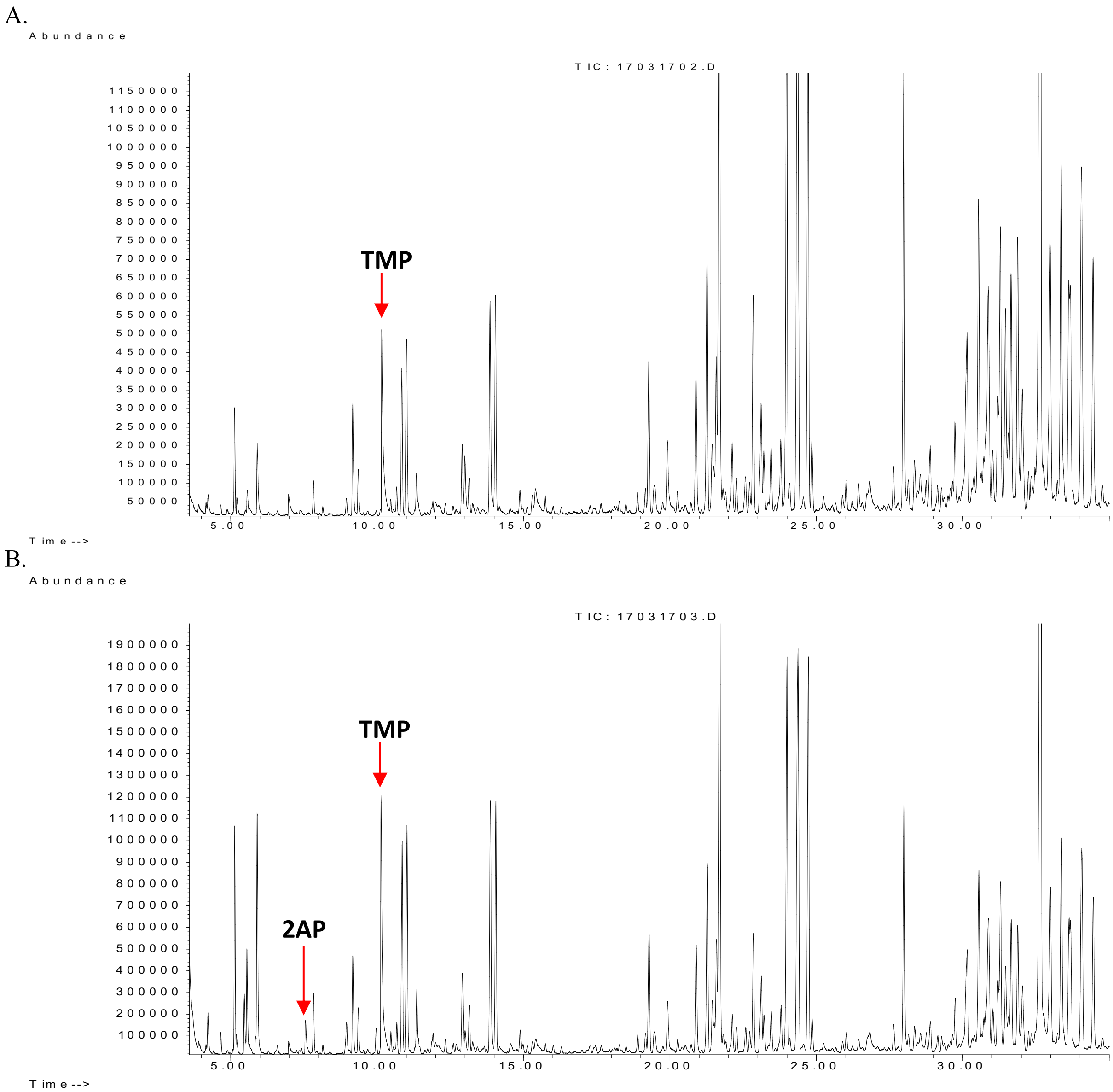

Supplementary Figure S1. GC-MS chromatograms of extracts from nonaromatic (A) and aromatic sponge gourd (B).

```
>lcl|c16191_g1_i1 len=2235 path=[1:0-2234]
CAATTTTCATAGATATCCGCTCGAGTGTGGTAAAATAGTCCATCGACTGAACCAAATTCGTAAAAAATTCAAAGATAAAA
TTTGCAGAAGAACAATCCATTACCAGTACGGACAAAATTCCGCTAAATAATTCGGAACTCAGCGTCTTTTTGTATATATA
TTGACATTGATCATCGCAGAGTCGATGCCATTTCCAATCTCACTAGTTTTCCCACGACATTGAAGAGAAGAGCTCTGTAA
AAATGGCGATTCCGATTCCCAGTCGGCAGCTATTCATCGGTGGCGAGTGGAGGGAACCGGTGCTCAAGAAACGCATCCCC
ATCGTCAACCCTGCTACTGAAGAAATAATCGGATCTATTCCCGCAGCTACTGCGGAAGATGTAGAGTTAGCTGTAGATGC
TGCTAGAAAAGCCCTTGCGAGGAATAAAGGCAAAGATTGGGCCTCTGCTTCGGGGGCTCTTCGTGCCAAGTATTTGCGTG
CTATTGCTGCTAAGATAACAGAGAGGAAATCAGAATTAGCGAAGCTTGAAACAATAGATTGTGGAAAACCTCTGGAAGAA
GCTGCATGGGACATCGATGATGTTGCTGGGTGCTTTGAATACTATGCGGATCTTGCTGAAGGGTTGGATGCAAAGCAAAA
AGCTCCTGTTTCCGTTCCCATGGATACGTTCAAGAGCTATGTTCTTAAAGAACCCATTGGAGTTGTTGGGTTGATTACTC
CTTGGAACTATCCTCTATTGATGGCTGTATGGAAAGTAGCACCTGCCTTGGCTGCTGGGTGTGCTGCAATATTGAAGCCG
TCAGAATTGGCATCTGTCACCTGTTTGGAACTAGCGGAAATTTGTAAAGATGTTGGTCTTCCACCTGGCATTTTGAATAT
TCTGACAGGATTGGGCCCTGAAGCTGGTGCTCCTCTAGCATCTCATCCTCATGTTGACAAGATTGCATTTACTGGGAGTG
GTGCTACTGGAAGCAAGATTATGACAGCAGCTGCTCAACTTGTCAAGCCAGTCACCATGGAACTTGGTGGAAAAAGTCCG
ATTGTTATTTTTGAGGATGTCGACCTTGATAAGGCTGCCGAATGGACGATCTTTGGTTGCTTTTGGACAAATGGTCAGAT
TTGCAGTGCCACATCTCGTCTAATTGTACACGAAAACATTGCTGATAAATTCTTGGATAAGCTCGTGCAATGGTGCAAGA
ACATTAAGATTTCAGATCCTTTGGAAGAAGGTTGCAGGCTTGGCCCTGTTGTTAGTGCAGGACAGTATGAGAAAGTATTG
AAGTTTGTCTCAACTGCTGAGAGCGAAGGTGCAAAGATTCTATTTGGTGGAGTTCGTCCTAAGCACCTAAACAAGGGATA
CTTCGTCGAACCAGCCATTATTACTAATGTTACAACCTCCATGCAAATATGGAGAGAAGAAGTCTTTGGACCTGTTCTAT
GTGCGAAGACTTTTAGTTCTGAAGATGAAGCAATTGAATTAGCAAATGATACGATATATGGGCTAGGTGCTGCTGTGATA
TCAAATGATTTAGAAAGGTGTGAGCGTGTAACCAAGGCTTTACAGGCAGGAATTGTGTGGGTTAATTGCTCGCAACCATG
CTTCACTCAAGCCCCATGGGGAGGCAACAAACGCAGTGGCTTTGGTCGAGAACTAGGGGAATGGGGACTTGAAAATTATC
TGACTGTAAAGCAGGTTACTCAATATGTATCCGATGAACCATGGGGATGGTACAAATCTCCTTCTAAACTGTAAAAGACA
CGAGCCTCTCCTCCGTCAAAAAGTTGCAGACACGTTCAGAATAAGCTCTGCCTTTGTCTACTGGAAGGAATAATCTGAGG
AAGAAAGTCTTGTACAACTAGTGCTGCTCTTTATTTCCTGGTTAGTTCAGTTAGGCTGCGTTTAAGAACTCGATTCAGTT
TCGAAATATCGTTGCTGAAAATATTAGATACTGTGACAATAGAAAATGCTCTAGGTAGCCTCAATGGGATTATCATATGA
TATGTCTGAGATTGTTGTATGAATTCGGGAAATAACTTTGAGCCTTTCTCTGTATTTCTTAATCTTGGCTTTGAAAATGT
GAGGAAAAACTTGGATTTCTTCTCAAGTGTTGCCCCTTTCTTGTGCTGTCATCGTATGCAGCTGCACACAGTTGGTATTT
TGTGGTTCCAATTAGGCGTTTTTGGTAAAATTATTGCAGATATTTTTTTAGTTCAATAATATTTGATTTTTTTGG
```

Supplementary Figure S2. Sequence of the contig c16191_g1_i1. Coding sequence (CDS) is highlighted in red.

```
 243 atggcgattccgattcccagtcggcagctattcatcggtggcgag
     M  A  I  P  I  P  S  R  Q  L  F  I  G  G  E
 288 tggagggaaccggtgctcaagaaacgcatccccatcgtcaaccct
     W  R  E  P  V  L  K  K  R  I  P  I  V  N  P
 333 gctactgaagaaataatcggatctattcccgcagctactgcggaa
     A  T  E  E  I  I  G  S  I  P  A  A  T  A  E
 378 gatgtagagttagctgtagatgctgctagaaaagcccttgcgagg
     D  V  E  L  A  V  D  A  A  R  K  A  L  A  R
 423 aataaaggcaaagattgggcctctgcttcgggggctcttcgtgcc
     N  K  G  K  D  W  A  S  A  S  G  A  L  R  A
 468 aagtatttgcgtgctattgctgctaagataacagagaggaaatca
     K  Y  L  R  A  I  A  A  K  I  T  E  R  K  S
 513 gaattagcgaagcttgaaacaatagattgtggaaaacctctggaa
     E  L  A  K  L  E  T  I  D  C  G  K  P  L  E
 558 gaagctgcatgggacatcgatgatgttgctgggtgctttgaatac
     E  A  A  W  D  I  D  D  V  A  G  C  F  E  Y
 603 tatgcggatcttgctgaagggttggatgcaaagcaaaaagctcct
     Y  A  D  L  A  E  G  L  D  A  K  Q  K  A  P
 648 gtttccgttcccatggatacgttcaagagctatgttcttaaagaa
     V  S  V  P  M  D  T  F  K  S  Y  V  L  K  E
 693 cccattggagttgttgggttgattactccttggaactatcctcta
     P  I  G  V  V  G  L  I  T  P  W  N  Y  P  L
 738 ttgatggctgtatggaaagtagcacctgccttggctgctgggtgt
     L  M  A  V  W  K  V  A  P  A  L  A  A  G  C
 783 gctgcaatattgaagccgtcagaattggcatctgtcacctgtttg
     A  A  I  L  K  P  S  E  L  A  S  V  T  C  L
 828 gaactagcggaaatttgtaaagatgttggtcttccacctggcatt
     E  L  A  E  I  C  K  D  V  G  L  P  P  G  I
 873 ttgaatattctgacaggattgggccctgaagctggtgctcctcta
     L  N  I  L  T  G  L  G  P  E  A  G  A  P  L
 918 gcatctcatcctcatgttgacaagattgcatttactgggagtggt
     A  S  H  P  H  V  D  K  I  A  F  T  G  S  G
 963 gctactggaagcaagattatgacagcagctgctcaacttgtcaag
     A  T  G  S  K  I  M  T  A  A  A  Q  L  V  K
1008 ccagtcaccatggaacttggtggaaaaagtccgattgttattttt
     P  V  T  M  E  L  G  G  K  S  P  I  V  I  F
1053 gaggatgtcgaccttgataaggctgccgaatggacgatctttggt
     E  D  V  D  L  D  K  A  A  E  W  T  I  F  G
1098 tgcttttggacaaatggtcagatttgcagtgccacatctcgtcta
     C  F  W  T  N  G  Q  I  C  S  A  T  S  R  L
1143 attgtacacgaaaacattgctgataaattcttggataagctcgtg
     I  V  H  E  N  I  A  D  K  F  L  D  K  L  V
1188 caatggtgcaagaacattaagatttcagatcctttggaagaaggt
     Q  W  C  K  N  I  K  I  S  D  P  L  E  E  G
1233 tgcaggcttggccctgttgttagtgcaggacagtatgagaaagta
     C  R  L  G  P  V  V  S  A  G  Q  Y  E  K  V
1278 ttgaagtttgtctcaactgctgagagcgaaggtgcaaagattcta
     L  K  F  V  S  T  A  E  S  E  G  A  K  I  L
1323 tttggtggagttcgtcctaagcacctaaacaagggatacttcgtc
     F  G  G  V  R  P  K  H  L  N  K  G  Y  F  V
1368 gaaccagccattattactaatgttacaacctccatgcaaatatgg
     E  P  A  I  I  T  N  V  T  T  S  M  Q  I  W
1413 agagaagaagtctttggacctgttctatgtgcgaagacttttagt
     R  E  E  V  F  G  P  V  L  C  A  K  T  F  S
1458 tctgaagatgaagcaattgaattagcaaatgatacgatatatggg
     S  E  D  E  A  I  E  L  A  N  D  T  I  Y  G
1503 ctaggtgctgctgtgatatcaaatgatttagaaaggtgtgagcgt
     L  G  A  A  V  I  S  N  D  L  E  R  C  E  R
1548 gtaaccaaggctttacaggcaggaattgtgtgggttaattgctcg
     V  T  K  A  L  Q  A  G  I  V  W  V  N  C  S
1593 caaccatgcttcactcaagccccatggggaggcaacaaacgcagt
     Q  P  C  F  T  Q  A  P  W  G  G  N  K  R  S
1638 ggctttggtcgagaactaggggaatggggacttgaaaattatctg
     G  F  G  R  E  L  G  E  W  G  L  E  N  Y  L
1683 actgtaaagcaggttactcaatatgtatccgatgaaccatgggga
     T  V  K  Q  V  T  Q  Y  V  S  D  E  P  W  G
1728 tggtacaaatctccttctaaactgtaa 1754
     W  Y  K  S  P  S  K  L  *
```

Supplementary Figure S3. Translated protein sequence of the transcript contig c16191_g1_i1.

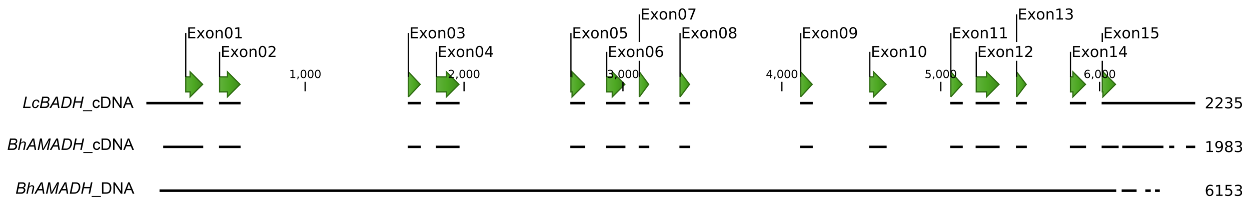

Supplementary Figure S4. Structure of *LcBADH* ortholog in sponge gourd compared with *BhAMADH* in winter melon

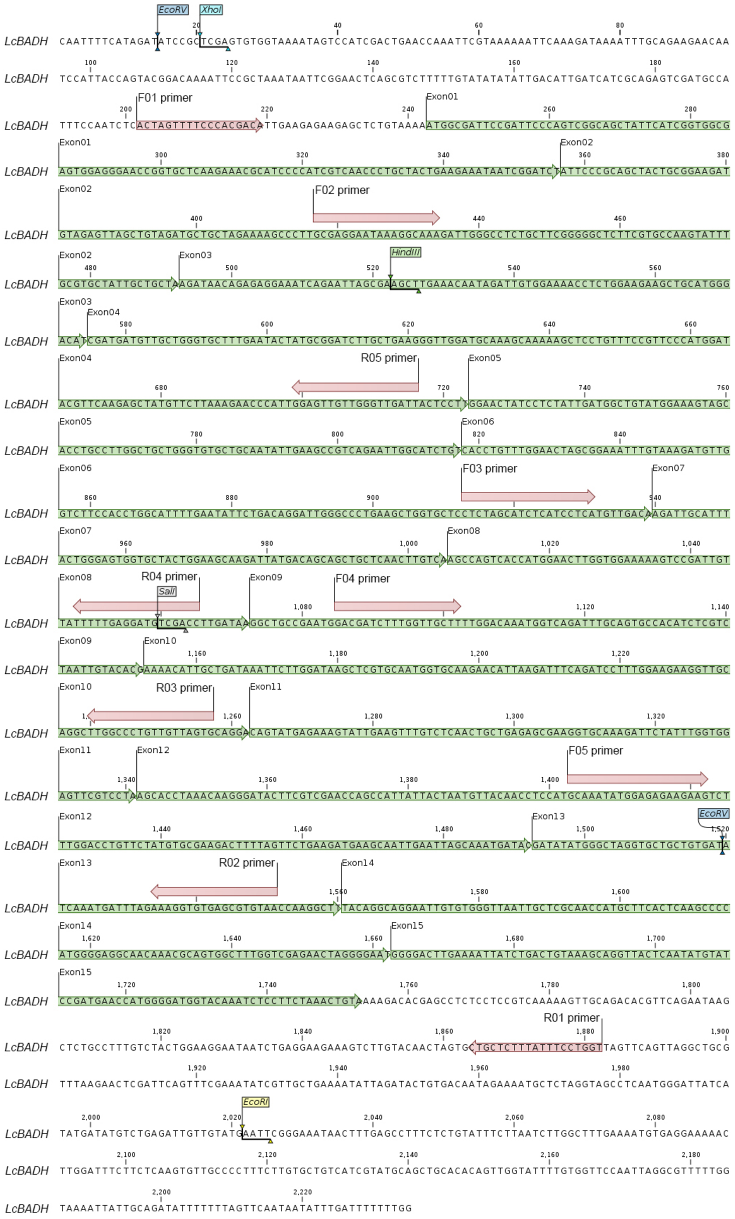

Supplementary Figure S5. Locations of primers on the *LcBADH* cDNA sequence.

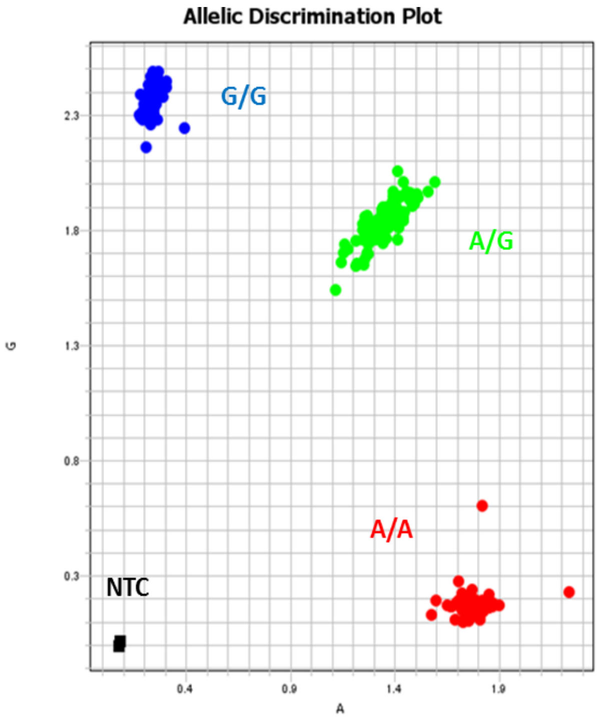

Supplementary Figure S6. Allelic discrimination plot of the three types of genotypes (A/A, G/G and A/G) based on *AroLuff* marker tested on total of 370 F_2 individuals.

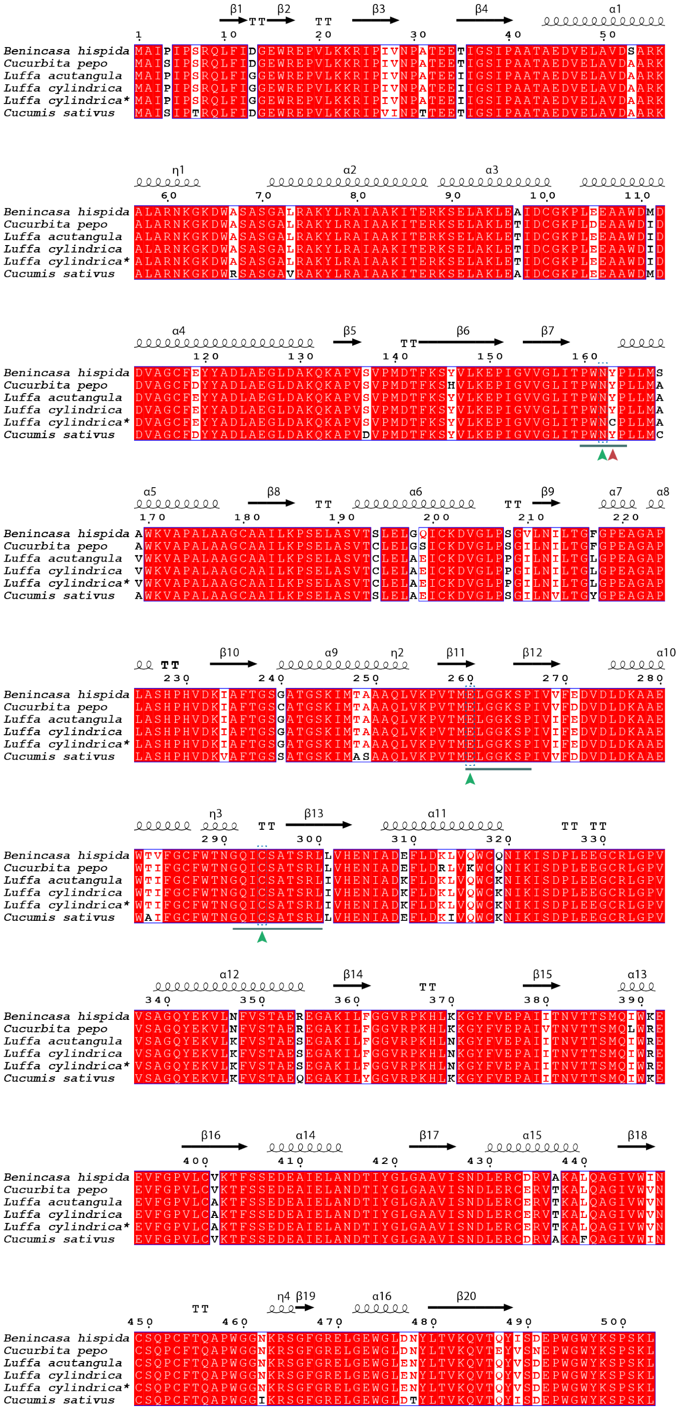

Supplementary Figure S7. Multiple protein sequence alignment of BADH/AMADH in *Benincasa hispida*, *Cucurbita pepo*, *Luffa acutangula*, *Luffa cylindrica* and *Cucumis sativus*. The aromatic *Luffa cylindrica* is denoted by an asterisk (*). Red shade stands for complete residue conservation. The bars under the alignment indicate the conserved catalytic sites in ALDH10 family. The most important redue in each catalytic site is highlighed by a dotted rectangular and indicated by a green arrowhead. The amino acid change in the aromatic sponge gourd (C163Y) is indicated by a red arrowhead.

Supplementary Table S1 *De novo* transcriptome assembly statistics of sponge gourd

**Before pre-processing**	
Number of raw reads	59,874,228
Read length (bp)	5,568,303,204
**Trinity assembly statistics**	
Number of contigs	63,392
Number of contigs containing CDS	50,146
Number of contigs containing BLAST hit	21,977
Contigs (bp)	66,075,847
N50 length (bp)	1880
Average contig length (bp)	1042.34
Median contig length (bp)	566

Supplementary Table S2 Genotypes and phenotypes of 370 F_2 progenies

F2 number	Genotype	Phenotype
1	G:G	Aromatic
2	A:A	Nonaromatic
3	A:G	Nonaromatic
4	A:G	Nonaromatic
5	A:G	Nonaromatic
6	A:G	Nonaromatic
7	A:A	Nonaromatic
8	A:G	Nonaromatic
9	A:G	Nonaromatic
10	A:G	Nonaromatic
11	A:G	Nonaromatic
12	A:A	Nonaromatic
13	A:A	Nonaromatic
14	A:A	Nonaromatic
15	A:G	Nonaromatic
16	A:G	Nonaromatic
17	A:A	Nonaromatic
18	A:G	Nonaromatic
19	G:G	Aromatic
20	A:G	Nonaromatic
21	A:G	Nonaromatic
22	A:A	Nonaromatic
23	A:A	Nonaromatic
24	A:A	Nonaromatic
25	G:G	Aromatic
26	A:G	Nonaromatic
27	A:A	Nonaromatic
28	A:G	Nonaromatic
29	A:G	Nonaromatic
30	G:G	Aromatic
31	A:G	Nonaromatic
32	A:G	Nonaromatic
33	G:G	Aromatic
34	A:A	Nonaromatic
35	A:G	Nonaromatic
36	G:G	Aromatic
37	A:A	Nonaromatic
38	A:G	Nonaromatic
39	A:G	Nonaromatic
40	A:A	Nonaromatic
41	G:G	Aromatic
42	G:G	Aromatic
43	A:G	Nonaromatic
44	A:A	Nonaromatic
45	A:G	Nonaromatic

F2 number	Genotype	Phenotype
46	A:G	Nonaromatic
47	A:A	Nonaromatic
48	G:G	Aromatic
49	A:A	Nonaromatic
50	G:G	Aromatic
51	G:G	Aromatic
52	A:G	Nonaromatic
53	G:G	Aromatic
54	G:G	Aromatic
55	G:G	Aromatic
56	A:G	Nonaromatic
57	A:G	Nonaromatic
58	A:A	Nonaromatic
59	A:G	Nonaromatic
60	A:G	Nonaromatic
61	A:G	Nonaromatic
62	G:G	Aromatic
63	A:A	Nonaromatic
64	G:G	Aromatic
65	A:G	Nonaromatic
66	A:A	Nonaromatic
67	A:G	Nonaromatic
68	A:A	Nonaromatic
69	A:G	Nonaromatic
70	A:A	Nonaromatic
71	A:G	Nonaromatic
72	A:A	Nonaromatic
73	A:G	Nonaromatic
74	A:G	Nonaromatic
75	A:G	Nonaromatic
76	A:G	Nonaromatic
77	A:G	Nonaromatic
78	A:A	Nonaromatic
79	A:A	Nonaromatic
80	A:A	Nonaromatic
81	G:G	Aromatic
82	A:G	Nonaromatic
83	A:A	Nonaromatic
84	A:G	Nonaromatic
85	A:G	Nonaromatic
86	A:A	Nonaromatic
87	A:G	Nonaromatic
88	A:G	Nonaromatic
89	G:G	Aromatic
90	A:G	Nonaromatic
91	A:G	Nonaromatic
92	A:G	Nonaromatic

F2 number	Genotype	Phenotype
93	A:G	Nonaromatic
94	A:G	Nonaromatic
95	G:G	Aromatic
96	G:G	Aromatic
97	G:G	Aromatic
98	A:G	Nonaromatic
99	A:G	Nonaromatic
100	G:G	Aromatic
101	A:A	Nonaromatic
102	G:G	Aromatic
103	A:A	Nonaromatic
104	A:G	Nonaromatic
105	A:G	Nonaromatic
106	A:G	Nonaromatic
107	A:G	Nonaromatic
108	A:A	Nonaromatic
109	G:G	Aromatic
110	A:A	Nonaromatic
111	G:G	Aromatic
112	G:G	Aromatic
113	A:G	Nonaromatic
114	A:G	Nonaromatic
115	G:G	Aromatic
116	A:G	Nonaromatic
117	A:G	Nonaromatic
118	G:G	Aromatic
119	G:G	Aromatic
120	A:G	Nonaromatic
121	G:G	Aromatic
122	G:G	Aromatic
123	A:A	Nonaromatic
124	G:G	Aromatic
125	A:G	Nonaromatic
126	A:G	Nonaromatic
127	A:G	Nonaromatic
128	A:G	Nonaromatic
129	A:G	Nonaromatic
130	A:G	Nonaromatic
131	G:G	Aromatic
132	A:A	Nonaromatic
133	A:A	Nonaromatic
134	G:G	Aromatic
135	A:G	Nonaromatic
136	A:G	Nonaromatic
137	A:A	Nonaromatic
138	A:A	Nonaromatic
139	A:G	Nonaromatic

F2 number	Genotype	Phenotype
140	A:A	Nonaromatic
141	G:G	Aromatic
142	A:G	Nonaromatic
143	G:G	Aromatic
144	A:G	Nonaromatic
145	G:G	Aromatic
146	A:G	Nonaromatic
147	A:G	Nonaromatic
148	A:G	Nonaromatic
149	A:A	Nonaromatic
150	A:A	Nonaromatic
151	A:G	Nonaromatic
152	G:G	Aromatic
153	A:G	Nonaromatic
154	G:G	Aromatic
155	A:G	Nonaromatic
156	A:A	Nonaromatic
157	A:A	Nonaromatic
158	A:G	Nonaromatic
159	A:A	Nonaromatic
160	G:G	Aromatic
161	A:G	Nonaromatic
162	A:A	Nonaromatic
163	G:G	Aromatic
164	A:G	Nonaromatic
165	A:G	Nonaromatic
166	A:A	Nonaromatic
167	A:G	Nonaromatic
168	A:G	Nonaromatic
169	G:G	Aromatic
170	A:G	Nonaromatic
171	G:G	Aromatic
172	A:A	Nonaromatic
173	A:G	Nonaromatic
174	G:G	Aromatic
175	A:A	Nonaromatic
176	A:A	Nonaromatic
177	A:A	Nonaromatic
178	A:A	Nonaromatic
179	A:G	Nonaromatic
180	A:G	Nonaromatic
181	A:G	Nonaromatic
182	A:G	Nonaromatic
183	A:G	Nonaromatic
184	A:G	Nonaromatic
185	A:G	Nonaromatic
186	G:G	Aromatic

F2 number	Genotype	Phenotype
187	A:G	Nonaromatic
188	A:G	Nonaromatic
189	A:G	Nonaromatic
190	A:G	Nonaromatic
191	G:G	Aromatic
192	A:G	Nonaromatic
193	A:G	Nonaromatic
194	A:G	Nonaromatic
195	A:G	Nonaromatic
196	G:G	Aromatic
197	A:A	Nonaromatic
198	A:A	Nonaromatic
199	A:G	Nonaromatic
200	A:G	Nonaromatic
201	A:A	Nonaromatic
202	A:A	Nonaromatic
203	A:A	Nonaromatic
204	A:G	Nonaromatic
205	A:G	Nonaromatic
206	G:G	Aromatic
207	G:G	Aromatic
208	G:G	Aromatic
209	G:G	Aromatic
210	G:G	Aromatic
211	G:G	Aromatic
212	A:G	Nonaromatic
213	A:A	Nonaromatic
214	A:G	Nonaromatic
215	G:G	Aromatic
216	G:G	Aromatic
217	A:A	Nonaromatic
218	A:A	Nonaromatic
219	A:G	Nonaromatic
220	G:G	Aromatic
221	A:G	Nonaromatic
222	G:G	Aromatic
223	A:A	Nonaromatic
224	A:G	Nonaromatic
225	A:A	Nonaromatic
226	A:G	Nonaromatic
227	A:A	Nonaromatic
228	A:A	Nonaromatic
229	A:A	Nonaromatic
230	A:G	Nonaromatic
231	A:G	Nonaromatic
232	A:G	Nonaromatic
233	G:G	Aromatic

F2 number	Genotype	Phenotype
234	A:G	Nonaromatic
235	A:A	Nonaromatic
236	A:A	Nonaromatic
237	G:G	Aromatic
238	A:G	Nonaromatic
239	A:A	Nonaromatic
240	A:A	Nonaromatic
241	A:A	Nonaromatic
242	A:G	Nonaromatic
243	A:G	Nonaromatic
244	G:G	Aromatic
245	A:G	Nonaromatic
246	A:G	Nonaromatic
247	A:G	Nonaromatic
248	A:A	Nonaromatic
249	A:G	Nonaromatic
250	A:A	Nonaromatic
251	A:A	Nonaromatic
252	A:A	Nonaromatic
253	A:A	Nonaromatic
254	G:G	Aromatic
255	A:G	Nonaromatic
256	A:G	Nonaromatic
257	A:G	Nonaromatic
258	A:A	Nonaromatic
259	A:G	Nonaromatic
260	G:G	Aromatic
261	G:G	Aromatic
262	A:G	Nonaromatic
263	G:G	Aromatic
264	G:G	Aromatic
265	G:G	Aromatic
266	A:G	Nonaromatic
267	A:G	Nonaromatic
268	A:G	Nonaromatic
269	A:G	Nonaromatic
270	A:A	Nonaromatic
271	A:A	Nonaromatic
272	A:G	Nonaromatic
273	G:G	Aromatic
274	A:G	Nonaromatic
275	G:G	Aromatic
276	A:A	Nonaromatic
277	A:G	Nonaromatic
278	A:G	Nonaromatic
279	A:G	Nonaromatic
280	A:G	Nonaromatic

F2 number	Genotype	Phenotype
281	A:A	Nonaromatic
282	G:G	Aromatic
283	A:G	Nonaromatic
284	A:G	Nonaromatic
285	A:A	Nonaromatic
286	G:G	Aromatic
287	A:G	Nonaromatic
288	A:G	Nonaromatic
289	G:G	Aromatic
290	G:G	Aromatic
291	A:G	Nonaromatic
292	A:G	Nonaromatic
293	G:G	Aromatic
294	A:G	Nonaromatic
295	A:A	Nonaromatic
296	A:G	Nonaromatic
297	A:G	Nonaromatic
298	A:G	Nonaromatic
299	G:G	Aromatic
300	G:G	Aromatic
301	A:G	Nonaromatic
302	G:G	Aromatic
303	A:G	Nonaromatic
304	A:G	Nonaromatic
305	A:A	Nonaromatic
306	A:G	Nonaromatic
307	A:G	Nonaromatic
308	A:G	Nonaromatic
309	A:G	Nonaromatic
310	A:G	Nonaromatic
311	A:G	Nonaromatic
312	A:A	Nonaromatic
313	A:G	Nonaromatic
314	A:A	Nonaromatic
315	G:G	Aromatic
316	A:G	Nonaromatic
317	A:G	Nonaromatic
318	A:A	Nonaromatic
319	G:G	Aromatic
320	A:G	Nonaromatic
321	G:G	Aromatic
322	A:G	Nonaromatic
323	A:A	Nonaromatic
324	G:G	Aromatic
325	A:G	Nonaromatic
326	A:G	Nonaromatic
327	A:G	Nonaromatic

F2 number	Genotype	Phenotype
328	A:G	Nonaromatic
329	A:G	Nonaromatic
330	G:G	Aromatic
331	A:A	Nonaromatic
332	A:G	Nonaromatic
333	A:G	Nonaromatic
334	A:A	Nonaromatic
335	A:G	Nonaromatic
336	A:G	Nonaromatic
337	A:G	Nonaromatic
338	A:G	Nonaromatic
339	A:G	Nonaromatic
340	A:G	Nonaromatic
341	A:G	Nonaromatic
342	A:G	Nonaromatic
343	A:A	Nonaromatic
344	A:G	Nonaromatic
345	A:G	Nonaromatic
346	A:G	Nonaromatic
347	A:G	Nonaromatic
348	A:G	Nonaromatic
349	G:G	Aromatic
350	A:G	Nonaromatic
351	A:G	Nonaromatic
352	A:A	Nonaromatic
353	A:G	Nonaromatic
354	A:A	Nonaromatic
355	A:A	Nonaromatic
356	A:A	Nonaromatic
357	A:G	Nonaromatic
358	A:G	Nonaromatic
359	G:G	Aromatic
360	A:A	Nonaromatic
361	A:A	Nonaromatic
362	A:A	Nonaromatic
363	G:G	Aromatic
364	A:G	Nonaromatic
365	G:G	Aromatic
366	A:G	Nonaromatic
367	G:G	Aromatic
368	G:G	Aromatic
369	A:G	Nonaromatic
370	A:G	Nonaromatic

Supplementary Table S3 Genotypes and phenotypes of BC_1F_2 interspecific cross.

No.	Code	Genotype	Phenotype
1	PB-00646_1	A:G	non-aromatic
2	PB-00647_1	A:A	non-aromatic
3	PB-00647_2	A:A	non-aromatic
4	PB-00648_1	A:A	non-aromatic
5	PB-00650_1	A:A	non-aromatic
6	PB-00650_2	A:A	non-aromatic
7	PB-00650_3	A:A	non-aromatic
8	PB-00650_4	A:A	non-aromatic
9	PB-00650_5	A:A	non-aromatic
10	PB-00650_6	A:A	non-aromatic
11	PB-00650_7	A:A	non-aromatic
12	PB-00650_8	A:A	non-aromatic
13	PB-00650_9	A:A	non-aromatic
14	PB-00650_10	A:A	non-aromatic
15	PB-00650_11	A:A	non-aromatic
16	PB-00650_12	A:A	non-aromatic
17	PB-00650_13	A:A	non-aromatic
18	PB-00651_1	A:A	non-aromatic
19	PB-00651_2	A:G	non-aromatic
20	PB-00653_1	A:G	non-aromatic
21	PB-00653_2	A:A	non-aromatic
22	PB-00653_3	A:A	non-aromatic
23	PB-00653_4	A:A	non-aromatic
24	PB-00653_5	A:A	non-aromatic
25	PB-00653_6	A:A	non-aromatic
26	PB-00654_1	A:A	non-aromatic
27	PB-00654_2	A:A	non-aromatic
28	PB-00654_3	A:A	non-aromatic
29	PB-00654_4	A:A	non-aromatic
30	PB-00655_1	A:A	non-aromatic
31	PB-00655_2	A:G	non-aromatic
32	PB-00656_1	A:G	non-aromatic
33	PB-00656_2	A:G	non-aromatic
34	PB-00656_3	A:G	non-aromatic
35	PB-00656_4	A:A	non-aromatic
36	PB-00656_5	A:G	non-aromatic
37	PB-00656_6	A:A	non-aromatic
38	PB-00656_7	A:A	non-aromatic
39	PB-00656_8	A:G	non-aromatic
40	PB-00656_9	G:G	aromatic
41	PB-00657_1	A:A	non-aromatic
42	PB-00657_2	A:A	non-aromatic
43	PB-00657_3	A:A	non-aromatic
44	PB-00657_4	A:A	non-aromatic
45	PB-00657_5	A:A	non-aromatic
46	PB-00657_6	A:A	non-aromatic
47	PB-00657_7	A:A	non-aromatic

No.	Code	Genotype	Phenotype
48	PB-00658_1	A:A	non-aromatic
49	PB-00658_2	A:G	non-aromatic
50	PB-00659_1	A:G	non-aromatic
51	PB-00659_2	A:G	non-aromatic
52	PB-00659_3	A:A	non-aromatic
53	PB-00659_4	G:G	aromatic
54	PB-00659_5	A:A	non-aromatic
55	PB-00659_6	G:G	aromatic
56	PB-00659_7	A:A	non-aromatic
57	PB-00659_8	A:G	non-aromatic
58	PB-00659_9	A:G	non-aromatic
59	PB-00659_10	G:G	aromatic
60	PB-00659_11	G:G	aromatic
61	PB-00659_12	A:G	non-aromatic
62	PB-00659_13	A:G	non-aromatic
63	PB-00660_1	A:A	non-aromatic
64	PB-00660_2	A:A	non-aromatic
65	PB-00660_3	A:A	non-aromatic
66	PB-00660_4	A:A	non-aromatic
67	PB-00660_5	A:A	non-aromatic
68	PB-00660_6	A:A	non-aromatic
69	PB-00660_7	A:A	non-aromatic
70	PB-00660_8	A:A	non-aromatic
71	PB-00660_9	A:A	non-aromatic
72	PB-00660_10	A:A	non-aromatic
73	PB-00660_11	A:A	non-aromatic
74	PB-00660_12	A:A	non-aromatic
75	PB-00660_13	A:A	non-aromatic
76	PB-00660_14	A:A	non-aromatic
77	PB-00660_15	A:A	non-aromatic
78	PB-00660_16	A:A	non-aromatic
79	PB-00660_17	A:A	non-aromatic
80	PB-00660_18	A:A	non-aromatic
81	PB-00660_19	A:A	non-aromatic
82	PB-00662_1	A:G	non-aromatic
83	PB-00662_2	A:A	non-aromatic
84	PB-00662_3	G:G	aromatic
85	PB-00662_4	A:A	non-aromatic
86	PB-00662_5	A:G	non-aromatic
87	PB-00662_6	A:G	non-aromatic
88	PB-00663_1	G:G	aromatic
89	PB-00663_2	A:G	non-aromatic
90	PB-00663_3	G:G	aromatic
91	PB-00663_4	A:A	non-aromatic
92	PB-00664_1	A:G	non-aromatic
93	PB-00664_2	A:G	non-aromatic

Supplementary Table S4 List of primers used for amplification of the full-length *LcBADH* gene

primer name	Sequences (5' – 3')
Forward F01	ACTAGTTTTCCCACGACA
Forward F02	GCGAGGAATAAAGGCAAA
Forward F03	CTCTAGCATCTCATCCTCA
Forward F04	GACGATCTTTGGTTGCTT
Forward F05	GCAAATATGGAGAGAAGAAG
Reverse R01	AATCAACCCAACAACTCC
Reverse R02	GGTCGACATCCTCAAAAA
Reverse R03	CACTAACAACAGGGCCAA
Reverse R04	TTACACGCTCACACCTTT
Reverse R05	ACCAGGAAATAAAGAGCAG

Supplementary Table S5 List of primers used in qRT-PCR analysis

Primer name	Sequences (5' – 3')
Actin_F	TTCACCATTCCGGTACCATT
Actin_R	TTTGCAACCGAGTGAGACTG
LcBADH_F	GGGAATGGGGACTTGAAAAT
LcBADH_R	GCAACTTTTTGACGGAGGAG

Supplementary Table S6 Identiy/similarity matrix of BADH gene translations in Cucurbitaceae. The upper and lower triangle of data represent the percent identity and the percent similarity, respectively. The asterisk (*) donotes the BADH of the aromatic *Luffa cylindrica*.

	B. hispida	*C. pepo*	*L. acutangula*	*L. cylindrica*	*L. cylindrica**	*C. sativus*
Benincasa hispida		97.41%	96.22%	96.22%	96.02%	96.42%
Cucurbita pepo	95.02%		96.22%	96.22%	96.02%	95.22%
Luffa acutangula	94.43%	94.43%		100%	99.80%	95.02%
Luffa cylindrica	94.43%	94.43%	100%		99.80%	95.02%
*Luffa cylindrica**	94.23%	94.23%	99.80%	99.80%		94.83%
Cucumis sativus	93.83%	91.84%	91.65%	91.65%	91.45%	
